# Nonlinear dispersive cell model for microdosimetry of nanosecond pulsed electric fields

**DOI:** 10.1038/s41598-020-76642-w

**Published:** 2020-11-10

**Authors:** Fei Guo, Lin Zhang, Xin Liu

**Affiliations:** Institute of Ecological Safety, Chongqing University of Posts and Telecommunications, Chongqing, 400065 China

**Keywords:** Permeation and transport, Biological models, Electrophysiology

## Abstract

For applications based on nanosecond pulsed electric fields (nsPEFs), the underlying transmembrane potential (TMP) distribution on the plasma membrane is influenced by electroporation (EP) of the plasma membrane and dielectric dispersion (DP) of all cell compartments which is important for predicting the bioelectric effects. In this study, the temporal and spatial distribution of TMP on the plasma membrane induced by nsPEFs of various pulse durations (3 ns, 5 ns unipolar, 5 ns bipolar, and 10 ns) is investigated with the inclusion of both DP and EP. Based on the double-shelled dielectric spherical cell model, the Debye equation describing DP is transformed into the time-domain form with the introduction of polarization vector, and then we obtain the time course of TMP by solving the combination of Laplace equation and time-domain Debye equation. Next, the asymptotic version of the Smoluchowski equation is included to characterize the EP of plasma membrane in order to observe more profound electroporation effects with larger pore density and electroporated areas in consideration of both DP and EP. Through the simulation, it is clearer to understand the relationship between the applied nsPEFs and the induced bioelectric effects.

## Introduction

Transmembrane potential (TMP) is induced on the plasma membrane when an external electric field is applied to a biological cell. In the case of TMP exceeding the supraphysiological range of the potential on the plasma membrane (0.4–1 V) with intense applied electric field, micro-pores appear on the membrane, and this phenomenon is called electroporation (EP)^1–3^. EP has become a common method for gene transfection, drug delivery, and has been studying for cancer treatment^4,5^.

Typically, EP is induced by pulsed electric fields with the field intensity of several kV/cm and the duration in the level of several hundred microseconds to several milliseconds^2,3^. Recently, electric pulses with the field intensity of several tens of kV/cm and duration in the level of nanoseconds have been regarded as a drug-free, non-thermal way to address cancer diseases^6,7^. Both model evidence and experimental results indicate that nanosecond pulsed electric fields (nsPEFs) induce structural and functional changes of intracellular organelles, which is different from traditional electroporation^8–10^. Unlike conventional EP, much more numerous but smaller-sized pores are created in almost all regions of the plasma membrane with the application of intense nsPEFs^11^, which cause a significant increase of conductivity of the plasma membrane during and after nsPEFs exposure^12,13^. The appearances of massive micro-pores and secondary effects are closely related to the distribution of TMP on the plasma membrane, therefore, evaluating of TMP on the plasma membrane accurately plays a critical role in predicting the desired biological effects^14,15^.

However, it is difficult to directly observe the time evaluation of TMP on the plasma membrane during nsPEFs exposure. The studies of exploring the relationship between nsPEFs and TMP commonly rely on theoretical analysis. In previous theoretical studies, two effects that were generally ignored can greatly affect the temporal and spatial distribution of TMP on the plasma membrane when a biological cell is exposed to the external nsPEFs. The first effect is the dielectric dispersion (DP) of all cell compartments, which means the conductivity and permittivity of each component of a biological cell are frequency-dependent, in consequence, TMP on the plasma membrane is largely influenced by the frequency spectrum of the applied nsPEFs^15–19^. The second effect is electroporation (EP) induced by intense external electric fields, significant increase in the conductivity of plasma membrane is observed during and after electroporation, and then the temporal and spatial distribution of TMP can be greatly affected by EP^20–24^. Traditionally, the Smoluchowski equation is used to investigate the creation and development of micro-pores on the plasma membrane when studying the effect of EP on TMP distribution^24^, and the effect of DP on TMP distribution has been investigated both in the time domain and frequency domain^15,17–20^. Merla et al.^17^ investigated the effects of both EP and DP on TMP with complex mathematics which involved DFT and IDFT, then the electric solution was coupled with an asymptotic electroporation model, but this algorithm involved a two-step process and cannot obtain the effects of both EP and DP on TMP distribution simultaneously. Joshi et al.^19^ presented the time-dependent TMP at the outer cell membrane with the introduction of both EP and DP based on the numerical distribution circuit approach. Salimi et al.^20^ investigated membrane dielectric dispersion in nanosecond pulsed electroporation of biological cells, based on a single-shelled cell model. Lately, Chiapperino et al.^21^ established a nonlinear, dispersive multiphysics model to simulate the EP and DP process of irregular cells.

In this study, an improved method based on Salimi et al.^20^, the effects of both DP and EP on TMP can be investigated simultaneously with the introduction of polarization vector, which is very convenient for us to investigate the temporal and spatial distribution of TMP based on the double-shelled cell model^22^. In addition, the temporal and spatial results induced by unipolar pulse and bipolar pulse are discussed based on the proposed model.

## Methods

### Dielectric double-shelled cell model

**Figure 1:**
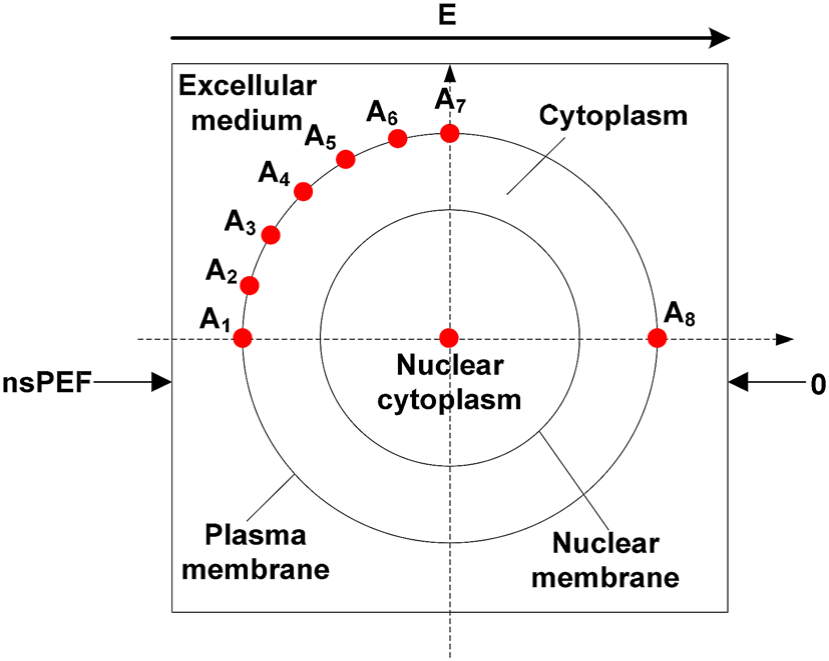
Dielectric double-shelled cell model.

The model containing a sphere with a smaller sphere inside is established as the dielectric double-shelled cell model and is adopted in our study, as shown in Fig. 1. The large and small spheres are all shielded by thin layers (represents the plasma membrane or nuclear membrane). Each component of this model is assumed to be isotropy. To analyze the evolution of pore density and TMP on the plasma membrane, seven sampling points (A_1_–A_7_) are selected, and the angle between every next two points is 15°. The geometrical parameters of this model are detailed in Table 1.

**Table 1:** Cell parameters used in our study.

Parameter type	Description/Symbol	Value
Geometrical parameters (μm)	Cell radius^18^	5
	Plasma membrane thickness^18^	0.01
	Nuclear radius^14^	2.5
	Nuclear membrane thickness^14^	0.01
Conductivity (S/m)	Extracellular^17^	0.55
	Plasma membrane^17^	1.1 × 10^–7^
	Cytoplasm^17^	0.55
	Nuclear membrane^16^	1.1 × 10^–5^
	Nuclear cytoplasm^14^	0.55
Relative permittivity	Extracellular^17^	67.00
	Plasma membrane^16^	5
	Cytoplasm^17^	67.00
	Nuclear membrane^14^	5
	Nuclear cytoplasm^14^	67.00
Relaxation parameters^16^	First relaxation time (*τ*_1_)	3.0 × 10^–9^ s
	Second relaxation time (*τ*_2_)	4.6 × 10^–10^ s
	First relaxation amplitude (Δ*ε*_1_)	2.3 × 10^–11^ F/m
	Second relaxation amplitude (Δ*ε*_2_)	7.4 × 10^–12^ F/m
	High frequency permittivity (*ε*_∞_)	13.9 × 10^–12^ F/m
Electroporation parameters^21^	Electroporation parameters (*α*)	1.0 × 10^9^ (m^2^ × s)^-1^
	Equilibrium pore density (*N*_0_)	1.5 × 10^9^ m^-2^
	Characteristic voltage (V_ep_)	0.258 V
	Electroporation constant (q)	2.46
	Pore radius (*r*_p_)	0.76 nm
	Energy barrier within pore (*w*_0_)	2.65
	Conductivity of aqueous pore (*σ*_p_)	1.3 S/m
	Relative entrance length of pores (n)	0.15
	Temperature (T)	295 K
	Universal gas constant (R)	8.314 J/K/*mol*
	Faraday’s constant (F)	9.65 × 10^4^ C/*mol*

### Debye equation

The static cell model is often treated as frequency-independent, and the cellular components should be regarded as lossy dielectrics when the applied electric field with frequency higher than megahertz. Commonly, effective conductivity and effective dielectric permittivity are used to describe the changes of dielectric parameters with frequency. Second-order Debye equation, which describes the complex permittivity, is used in calculation of TMP in time domain. The equation is expressed as:

1 $$ \varepsilon (\omega ) = \varepsilon_{\infty } + \frac{{\Delta \varepsilon_{1} }}{{1 + j\omega \tau_{1} }} + \frac{{\Delta \varepsilon_{2} }}{{1 + j\omega \tau_{2} }} $$

For a linear and isotropic medium the polarization vector is expressed as:

2 $$ P = (\varepsilon - \varepsilon_{0} )E $$

where *ε* and *ε*_0_ are the permittivity of the medium and vacuum, respectively. Dispersion is transformed into the time-domain form by defining the polarization of the medium as a function of the electric field and its time derivatives. For a second order dispersive medium, substitution of Eq. (1) into Eq. (2) taking *jω* with the derivative with respect to time yields is expressed as:

3 $$ \begin{aligned} & P + (\tau_{1} + \tau_{2} )\frac{\partial P}{{\partial t}} + \tau_{1} \tau_{2} \frac{{\partial^{2} P}}{{\partial t^{2} }} = (\varepsilon_{{{\text{m}}0}} - \varepsilon_{0} )E \\ & \quad + \left[ {\left( {\varepsilon_{{{\text{m}}0}} - \Delta \varepsilon_{1} - \varepsilon_{0} } \right)\tau_{1} + \left( {\varepsilon_{{{\text{m}}0}} - \Delta \varepsilon_{2} - \varepsilon_{0} } \right)\tau_{2} } \right]\frac{\partial E}{{\partial t}} \\ & \quad + \left( {\varepsilon_{{{\text{m}}0}} - \Delta \varepsilon_{1} - \Delta \varepsilon_{2} - \varepsilon_{0} } \right)\tau_{1} \tau_{2} \frac{{\partial^{2} E}}{{\partial t^{2} }} \\ \end{aligned} $$

where *ε*_m0_ is the low frequency permittivity of the membrane.

4 $$ \varepsilon_{{{\text{m}}0}} = \varepsilon_{\infty } + \Delta \varepsilon_{1} + \Delta \varepsilon_{2} $$

### Electroporation equation

The EP model used here is the asymptotic version of the Smoluchoski equation, and this model is plausible for signal durations in the nanosecond time scale^25^. Equation 5 describes the rate of creation and destruction of hydrophilic membrane pores per local membrane area *N*(*t*) as a function of the TMP(*t*).

5 $$ \frac{{{\text{d}}N(t)}}{{{\text{d}}t}} = \alpha e^{{\left( {1 - q} \right)\left( {\frac{{{\text{TMP}}(t)}}{{{\text{V}}_{{{\text{ep}}}} }}} \right)^{2} }} \left( {e^{{q\left( {\frac{{{\text{TMP}}(t)}}{{{\text{V}}_{{{\text{ep}}}} }}} \right)^{2} }} - \frac{N(t)}{{N_{0} }}} \right) $$

where TMP(*t*) is the TMP on the plasma membrane or the nuclear membrane. The conductivity *σ*_m_ of the plasma membrane or the nuclear membrane in EP is described as ^21^:

6 $$ \sigma_{{\text{m}}} (t) = \sigma_{{{\text{m0}}}} + N(t)\pi r_{{\text{p}}}^{2} \sigma_{{\text{p}}} K $$

where *σ*_m0_ is the initial conductivity of the membrane, the expression of *K* can be found in ^21^.

Equations (1)–(6) are all calculated on the domain of plasma membrane and nuclear membrane. The definitions and typical values of the constants in those equations are given in Table 1.

### Features of the nsPEFs

**Figure 2:**
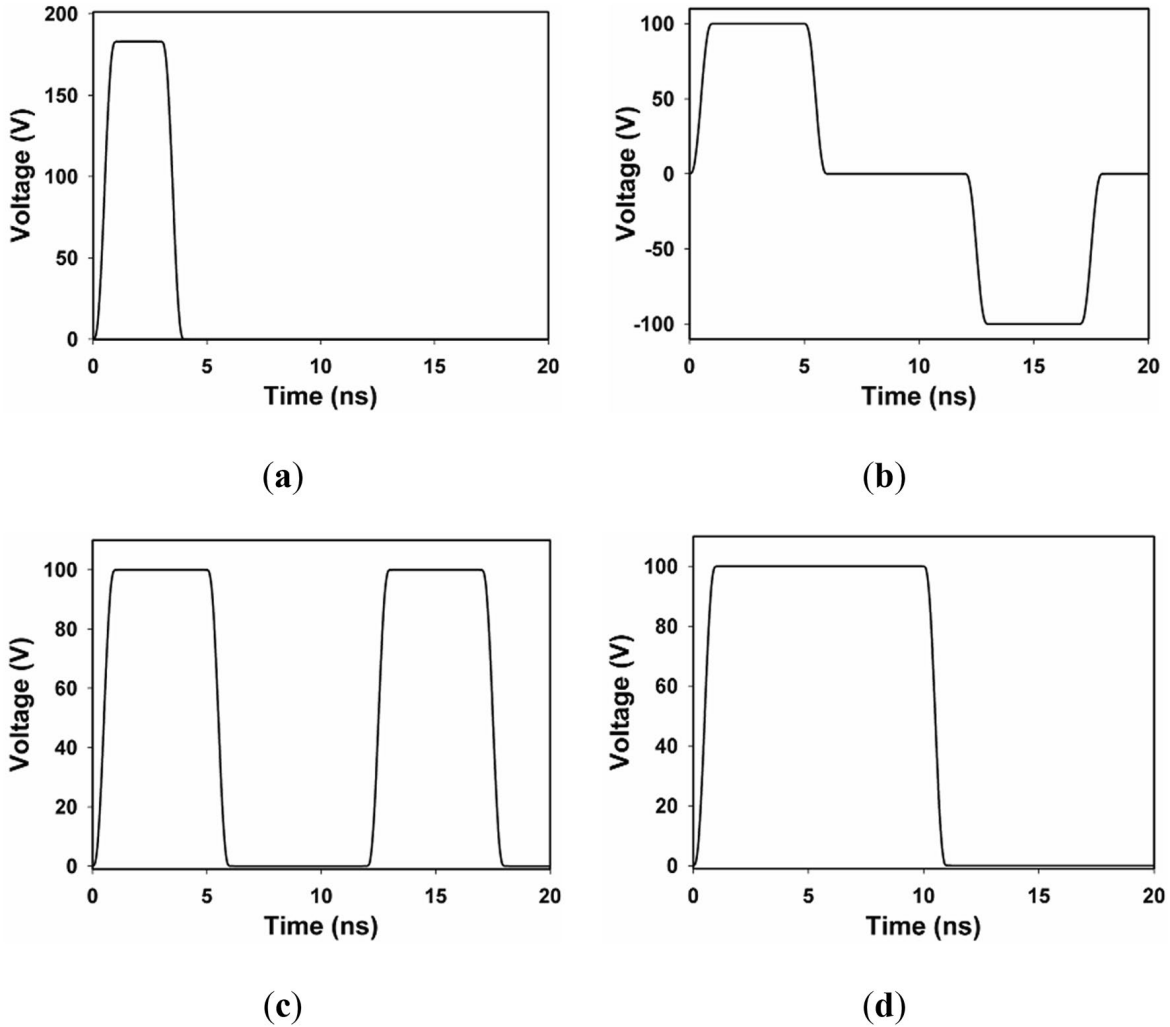
Electrical pulses with various pulse durations, magnitudes, and polarities. (**a**) nsPEFs of duration of 3 ns, (**b**) bipolar nsPEFs of pulse duration of 5 ns with time interval of 6 ns, (**c**) unipolar nsPEFs of pulse duration of 5 ns with time interval of 6 ns, (**d**) nsPEFs of duration of 10 ns. For 3 ns pulse, the ratio of voltage to distance is 18.3 kV/cm (183 V/100 μm), and for the latter three pulses, which is 10 kV/cm (100 V/100 μm), to ensure the same power density within all cases for comparison.

Trapezoidal-shaped pulses are adopted, as suggested in^16^. The pulse durations are 10 ns and 3 ns with field intensity of 10 and 18.3 kV/cm, respectively. In addition, bipolar pulse with pulse duration of 5 ns and interval of 6 ns, and unipolar pulse with pulse duration of 5 ns and interval of 6 ns are adopted both with field intensity of 10 kV/cm. All pulses have the same power density to obtain comparable results. The rise and fall times are chosen to equal to 1 ns for all pulses (Fig. 2).

### Model settings and calculation of the induced TMP

The calculations are performed in COMSOL Multiphysics 5.3a using the Electric currents and the PDE modes-coefficient form. The opposite vertical faces of the block are modeled as electrodes, which is done by assigning electric potential to each face. The left electrode is set to the above electric pulses and the right is connected to the ground to obtain the desired electric field. The remaining faces of the block are modeled to be insulating. The mesh size is refined until there is less than a 2% difference in the field results between refinements, resulting in fine mesh setting. The electric potential *φ* inside and outside the cell is then computed by solving the equation.

7 $$ \frac{{ - \nabla \cdot \partial (\varepsilon_{0} \nabla \varphi + P)}}{\partial t} - \nabla \cdot \sigma_{{\text{m}}} (t)\nabla \varphi = 0 $$

We use Electric Currents to solve the Laplace equation, the PDE modes-coefficient form to solve the EP equation and the time-domain Debye equation. The Laplace equation is solved at the sub-domains of extracellular medium, plasma membrane, cytoplasm, nuclear membrane and nuclear cytoplasm, the EP equation is solved inside the sub-domain of plasma membrane, and the time-domain Debye equation is solved inside the sub-domains of plasma membrane and nuclear membrane, the initial value of all the variables are set to zero at *t* = 0 except for the initial density of the pores of the plasma membrane is set to *N*_0_. Finally, the induced TMP is calculated as the difference between electric potentials on both sides of the membrane:

8 $$ \Delta \varphi = \varphi_{{\text{o}}} (t) - \varphi_{{\text{i}}} (t) $$

## Results

### Simulation verification

**Figure 3:**
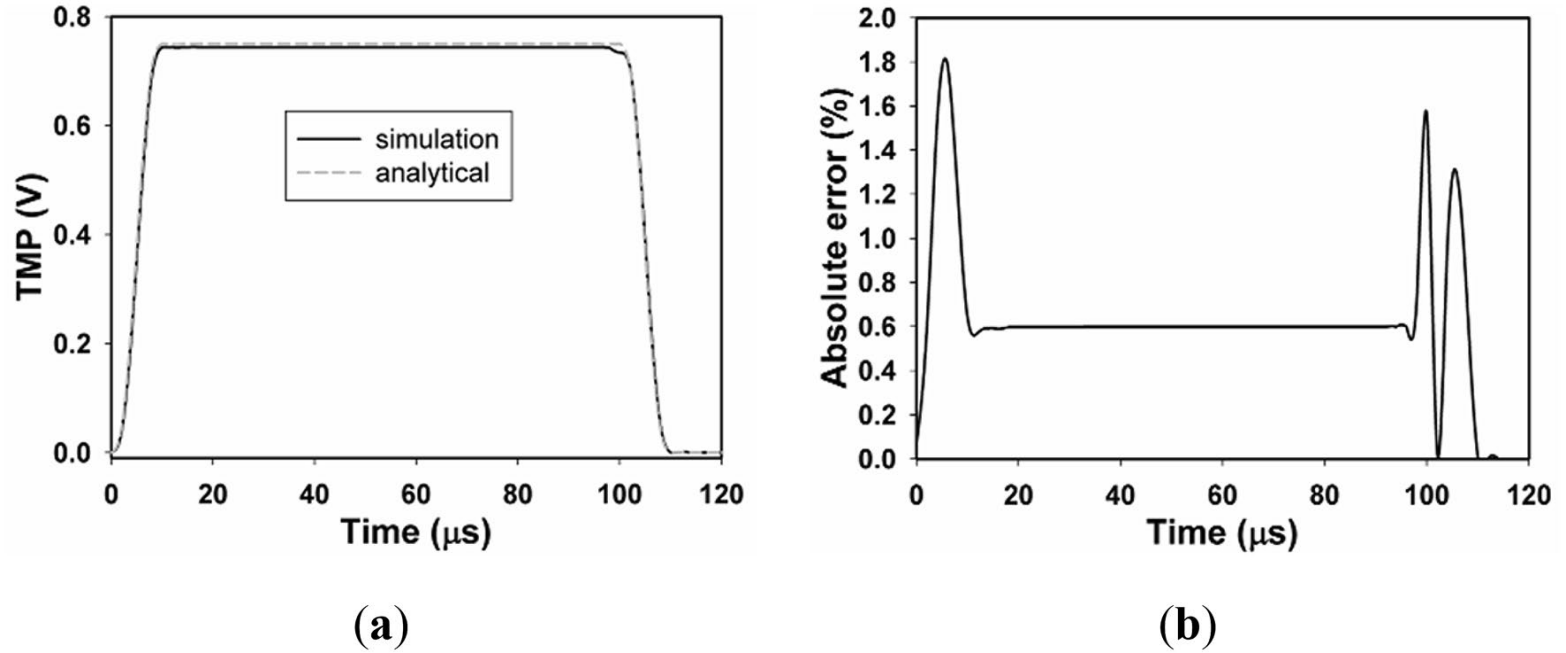
Time evolution of TMP of point A_1_. (**a**) Time evolution of TMP of A_1_ with μsPEF of 100 μs and 1 kV/cm, (**b**) the absolute error between analytical and simulation results, and the analytical result is obtained by solving the second-order Schwan equation.

To test the accuracy of the Comsol Multiphysics code, based on a static dielectric cell model without neither DP nor EP, we examine the TMP of point A_1_ (where TMP is maximum) with the electric field of pulse duration of 100 μs and field intensity of 1 kV/cm by comparing the analytical and simulation results. The analytical result is done by solving the second-order Schwan equation^26^ with parameters in Table 1. Figure 3 shows the time evolution of TMP of point A_1_, and the simulation result agrees very well with the analytical result, yet, the analytical result is a bit larger between 5 and 105 μs, which could be due to the uneven electric field formed by the limited ratio of electrode plate length to plate spacing in the simulation model. By reducing the ratio of the length of the electrode plate to the distance between the plates, the error can be reduced, but the simulation time will also be prolonged. In general, the temporal trend of the simulation and analytical results is similar, so the simulation has a satisfactory accuracy. The reason why we use the static cell model is that the analytical results of TMP in the cell model with either DP or EP are complicated. Furthermore, electric field with pulse duration of 100 μs instead of 10 ns is used here because the time course of TMP induced by nsPEFs with pulse duration less than the charging time of plasma membrane (~ 1 μs) is complicated.

### TMP distribution with and without DP

**Figure 4:**
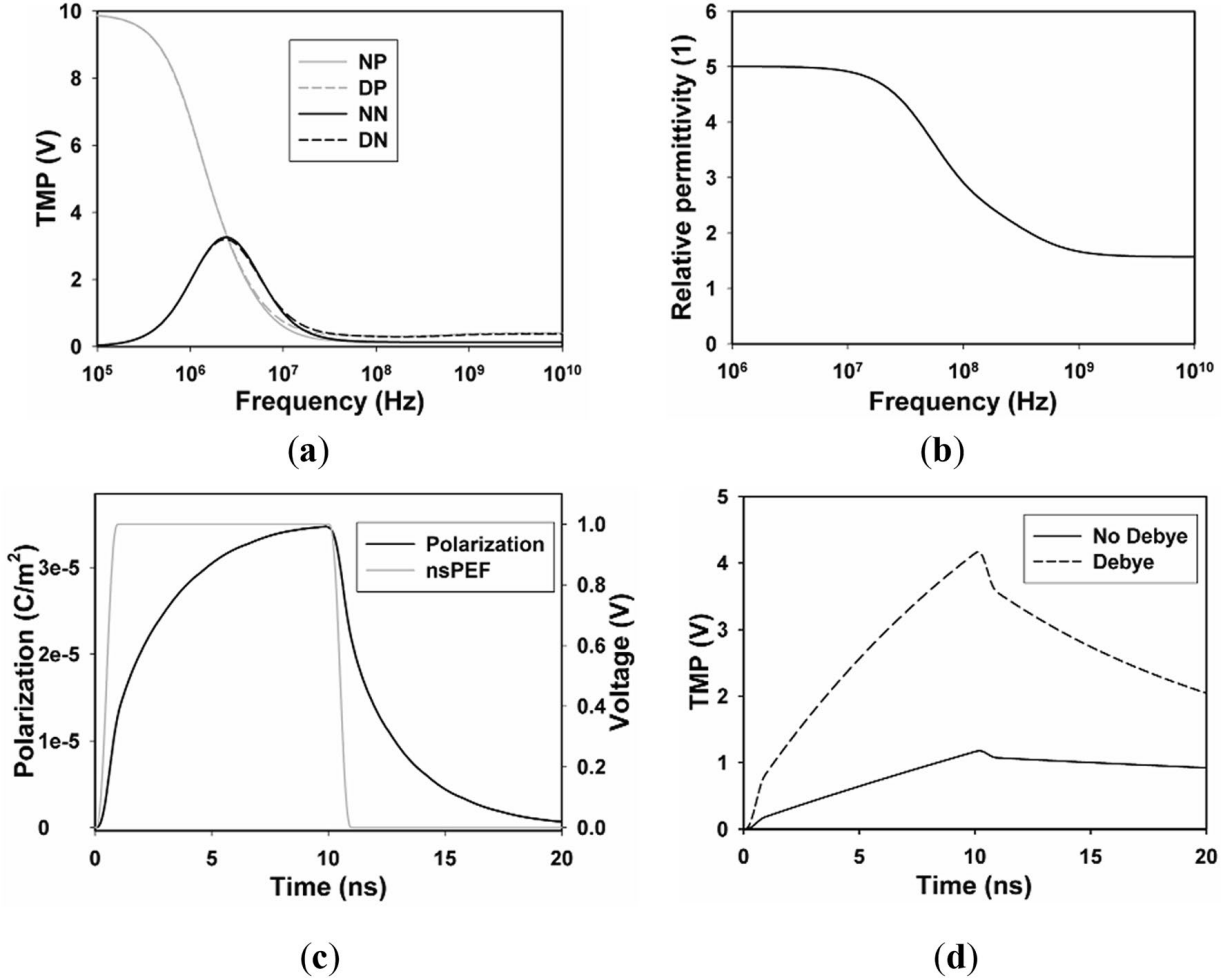
TMP distribution with and without DP. (**a**) The induced TMP on the cellular membrane (NP for non-dispersive plasma membrane, DP for dispersive plasma membrane) and nuclear membrane (NN for non-dispersive nuclear membrane, DN for dispersive nuclear membrane) versus frequency when the amplitude of the electric field is 10 kV/cm, (**b**) relative permittivity of plasma membrane versus frequency, (**c**) time courses of nsPEFs (gray) and polarization of point A_1_ (black), (**d**) time courses of TMP of points A_1_ in dispersive (dotted line) and non-dispersive (solid line) mode.

First, we investigate TMP distribution on the plasma membrane and nuclear membrane in frequency domain with two different modes, namely, cell model with and without DP, and the results are shown in Fig. 4a. TMP on the plasma membrane shows first-order low-pass filter characteristics, while TMP on the nuclear membrane shows first-order band-pass filter characteristic approximately, which agrees well with previous studies^27^.

TMP distribution with the introduction of DP is compared with those without DP, and it indicates that TMP is underestimated with the pulse frequency above 10^6^ Hz when DP is not taken into account. The relative permittivity of plasma membrane starts to decrease from 10^6^ Hz, then reaches its high-frequency value (*ε*_∞_/*ε*_0_, *ε*_0_ is the permittivity of vacuum) of about 1.57 at 10^10^ Hz (Fig. 4b), where the biggest difference between TMP on the plasma membrane with and without DP is observed.

With the definition of polarization vector P, the second-order Debye equation which describes dielectric relaxation of plasma membrane and nuclear membrane in the frequency domain is transformed into the time-domain form by Laplace transform, and then TMP distribution which includes DP with the application of nsPEFs can be solved in the time domain. The time course of polarization vector of point A_1_ with the application of nsPEFs (pulse duration of 10 ns, filed intensity of 10 kV/cm, rise time of 1 ns) is shown in Fig. 4c. The polarization vector changes rapidly during the rising and decreasing periods of the applied nsPEFs, and achieves its peak value of about 3.5 × 10^–5^ C/m^2^ at 12.9 ns, corresponding to a relative permittivity of 5 (equals to static relative permittivity of plasma membrane), which can prove the correctness of our simulation.

The time courses of TMP of point A_1_ with and without DP are shown in Fig. 4d. TMP of plasma membrane is always larger with DP than those without during our limited observation time, and the biggest difference is about 3 V. The simulation results are in well agreement with previous studies^17–19^, which indicates that TMP is underestimated when the DP is not taken into account, in other words, temporal and spatial distribution of TMP can be obtained more accurately with the inclusion of dielectric relaxation of all cell compartments.

### Temporal results with and without a nucleus

**Figure 5:**
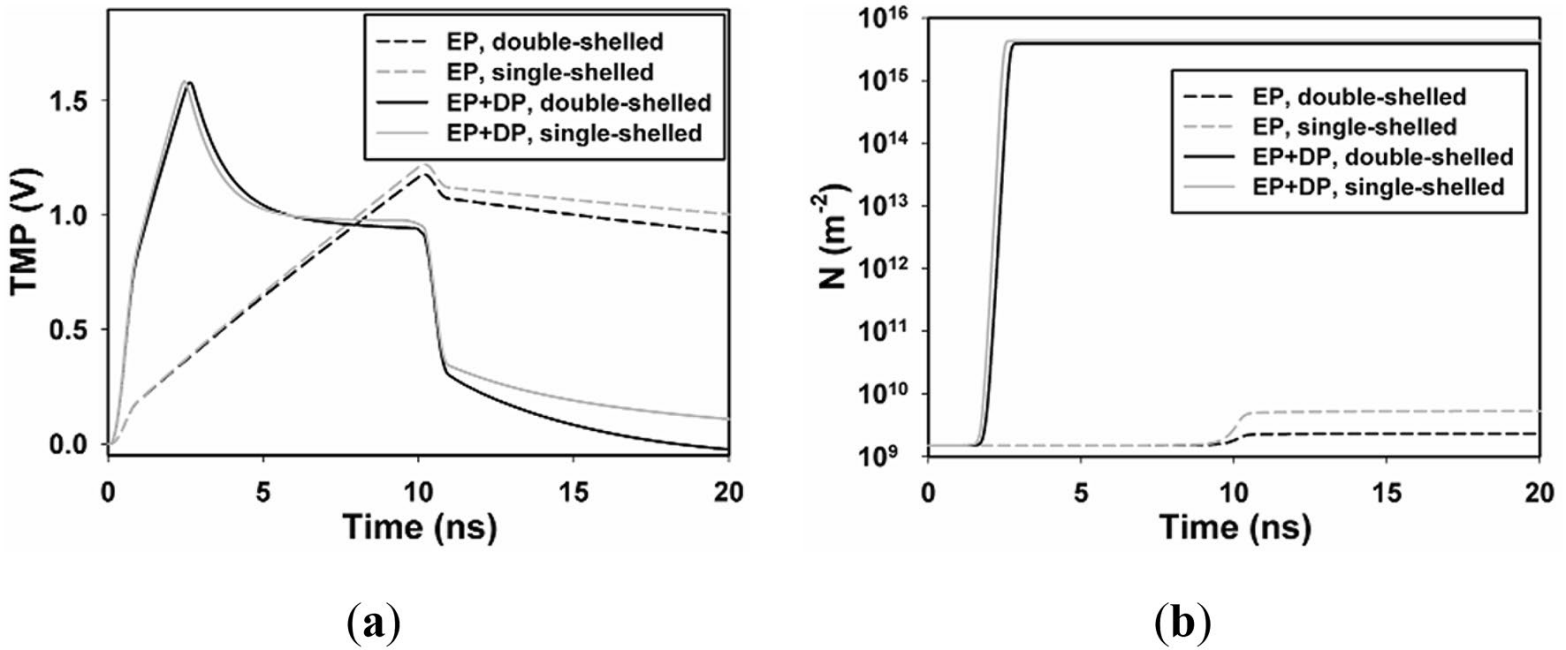
Temporal results with and without nucleus of point A_1_. (**a**) Time evolution of TMP of A_1_, (**b**) time evolution of N of A_1_ with nsPEF of 10 ns and 10 kV/cm. The black lines represent the results of double-shelled cell model, and the gray ones represent the results of single-shelled cell model.

The double-shelled model proposed in this paper is based on Kotnik et al.^22^, but dielectric relaxation is not introduced in their model. We compared the temporal distribution of TMP and pore density at point A_1_ with and without a nucleus (double-shelled and single-shelled cell model) when the cell exposed to nsPEF (pulse duration of 10 ns, filed intensity of 10 kV/cm, rise time of 1 ns). As shown in Fig. 5, the simulation result of double-shelled model without DP is consistent with that of Kotnik et al. And in the single-shelled model, the trend of TMP is consistent with that of Salimi et al.^20^ while the response time of TMP obtained by double-shelled model was a little shorter than that of single-shelled model (about 0.2 ns) whether DP was considered or not, and the TMP of the double-shelled model was slightly larger than that of the single-shelled model after the pulse. The response time and stability of the double-shelled model were faster and larger. The induced voltage on the cell membrane in electric field is depended on not only the electric field intensity but also the dielectric constant of cell materials^26^. Therefore, it is predicted that the TMP on the cell membrane will be affected when the nucleus is considered, which means that it is more accurate to obtain the parameters of EP on the cell membrane by considering the internal structure of the cell.

### Temporal and spatial results with both EP and DP

**Figure 6:**
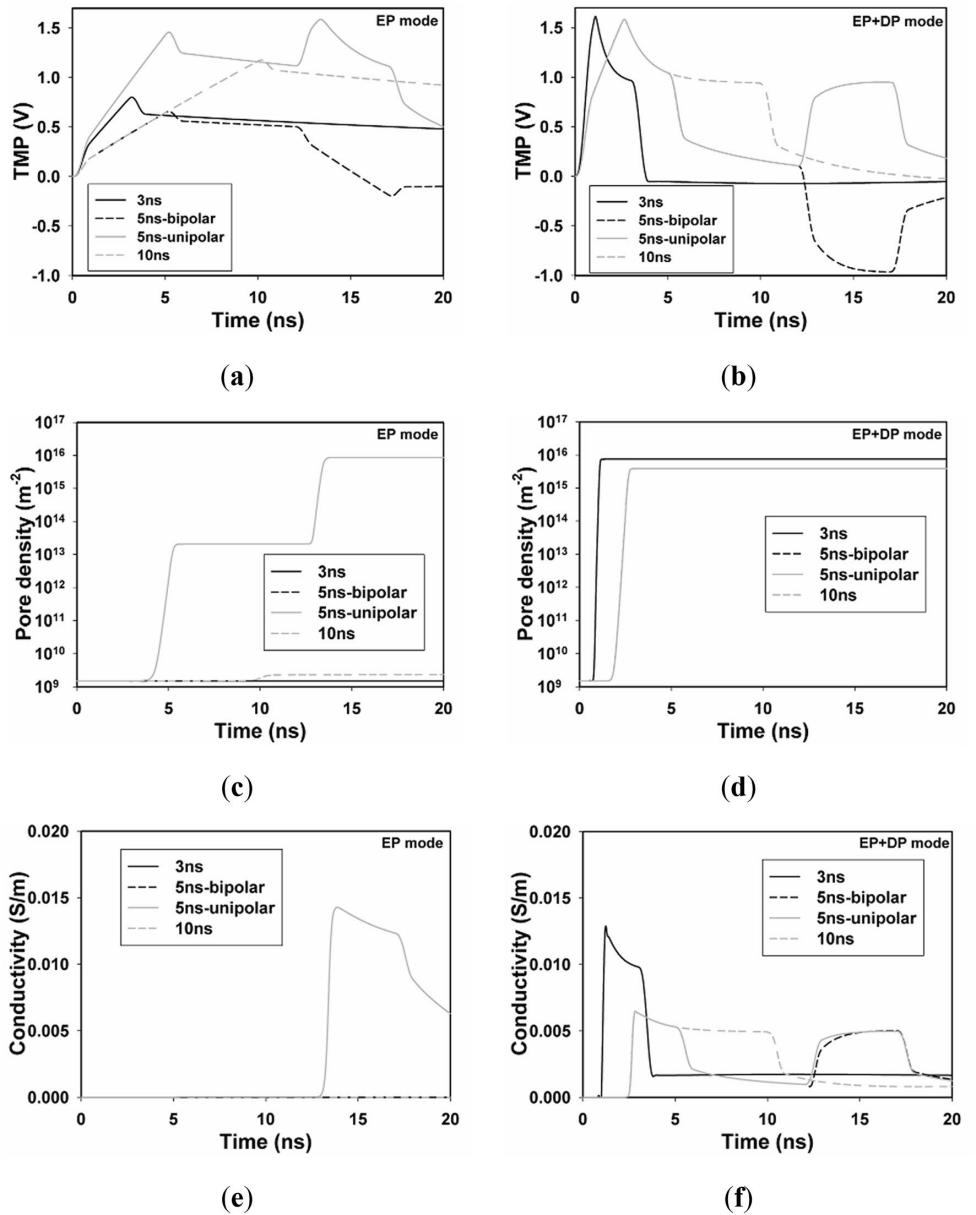
Time evolution of TMP and pore density with the application of four different nsPEFs. Temporal distribution of TMP of A_1_ in (**a**) EP mode and (**b**) EP + DP mode, pore density of A_1_ in (**c**) EP mode and (**d**) EP + DP mode, conductivity of A_1_ in (**e**) EP mode and (**f**) EP + DP mode, with four different nsPEFs.

In order to investigate the effects of both DP and EP on the temporal and spatial distribution of TMP on the plasma membrane, four nsPEFs with various pulse durations, field intensities, and polarities but with the same power density are selected to obtain comparable results. Time evolution of TMP and pore density of A_1_ with the application of the above four different nsPEFs in two different modes (EP and DP + EP) is shown in Fig. 6, TMP of A_1_ exceeds the critical threshold (1 V) with the application of 10 ns and 5 ns unipolar pulses, however, only the latter pulse induces a profound increase in pore density, which reaches the electroporation threshold (PT = 10^15^), in the EP only mode.

TMP and pore density of A_1_ reach their threshold values with all four nsPEFs in the DP + EP mode, and the time required to reach the threshold is much shorter than that of the EP only mode, with is in agreement with^17–19^.

After the electroporation threshold PT is overcome, the conductivity starts to increase. A significant increase in conductivity is observed with only the 5 ns unipolar pulse in EP only mode, while a significant increase in conductivity is observed with all four nsPEFs in the DP + EP mode.

**Figure 7:**
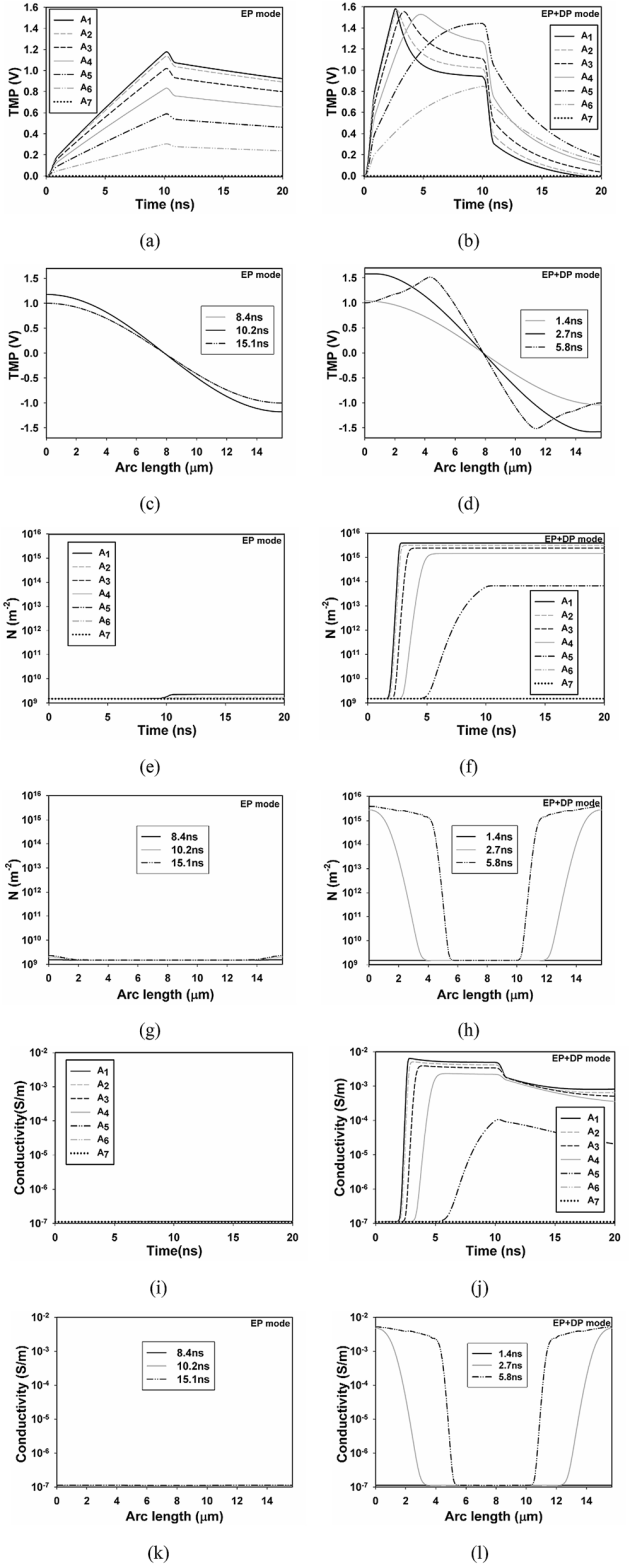
Temporal and spatial results from point A_1_ to A_7_. Temporal distribution of TMP of A_1_–A_7_ in (**a**) EP mode and (**b**) EP + DP mode, pore density of A_1_–A_7_ in (**e**) EP mode and (**f**) EP + DP mode, and conductivity of A_1_–A_7_ in (**i**) EP mode and (**j**) EP + DP mode, spatial distribution of TMP along the arc length of plasma membrane from A_1_ to A_8_ at different times in (**c**) EP mode and (**d**) EP + DP mode, pore density in (**g**) EP mode and (**h**) EP + DP mode, and conductivity in (**k**) EP mode and (**l**) EP + DP mode when nsPEFs of 10 ns and 10 kV/cm is applied.

To get in-depth understanding of the effects of both EP and DP on the temporal and spatial distribution of TMP, we select seven points separated by 15° in the upper left quarter on the plasma membrane to study the time course of TMP and pore density with the 10 ns pulse. And spatial distribution of TMP and pore density is examined along the half arc length of plasma membrane from A_1_ to A_8_, both in two different modes (EP and DP + EP). In the EP mode (Figs. 7a, c, e, and g), the TMP of A_1_ begins to increase at 0 ns when the pulse is delivered to the cell, exceeding a TMP threshold of about 1 V at 8.4 ns, then reaching its peak value of about 1.2 V at 10.2 ns, in agreement with^17^. The time trend is similar in A_2_–A_7_ except with a smaller TMP value, and the peak values of TMPs in A_1_–A_3_ exceed 1 V, while in A_4_–A_7_ those are smaller than 1 V.

Once the threshold of 1 V is overcome the pore density starts to increase, in accordance with^17^, however, pore density of A_1_ dose not reach up to the threshold (PT) of 10^15^ m^-2^ in our simulation, which may due to the differences in model parameters used in our simulation to those of^17^. Spatial distribution of TMP and pore density along the half arc length of plasma membrane gives similar results, and typical values are listed in Table 2. In addition, the significant increase in conductivity of A_1_–A_7_ along the arc length of plasma membrane at different times is not observed in the EP mode (Figs. 7i, k), and the results are in good agreement with the pore density distribution, demonstrating that cell is not effectively electroporated in the only EP mode.

**Table 2:** Typical values obtained from Fig. 7, which includes peak value of TMP and flat top value of pore density at different points, in both EP and DP + EP modes, and the time required to attain the typical values is also taken into account. Commonly, TMP of 1 V and pore density of 10^15^ are used as threshold values to predict the onset of EP, respectively.

Points	Peak value of TMP (EP)/time required to reach the peak value	Peak value of TMP (EP + DP)/time required to reach the peak value	Flat top value of pore density (EP)	Flat top value of pore density (EP + DP)
A_1_	1.18 V/10.2 ns	1.58 V/2.7 ns	2.3 × 10^9^/10.7 ns	3.90 × 10^15^/3.1 ns
A_2_	1.14 V/10.2 ns	1.57 V/2.8 ns	1.7 × 10^9^/10.7 ns	3.11 × 10^15^/3.4 ns
A_3_	1.02 V/10.2 ns	1.55 V/3.4 ns	*N*_0_	2.39 × 10^15^/4.1 ns
A_4_	0.83 V/10.2 ns	1.53 V/4.8 ns	*N*_0_	1.44 × 10^15^/6.6 ns
A_5_	0.59 V/10.2 ns	1.44 V/9.9 ns	*N*_0_	6.67 × 10^15^/10.5 ns
A_6_	0.30 V/10.2 ns	0.85 V/10 ns	*N*_0_	*N*_0_
A_7_	0	0	*N*_0_	*N*_0_

With the inclusion of both EP and DP in the cell model, the TMP of A_1_ starts to increase at 0 ns when the pulse is delivered to the cell, rapidly exceeding the TMP threshold of about 1 V at 1.4 ns, then reach its peak value of about 1.58 V at 2.7 ns. Relative faster change and larger value of TMP are achieved with the inclusion of DP (Figs. 7b, d). Once the threshold of 1 V is overcome, the pore density begins to increase to reach the membrane electroporation threshold of 10^15^ m^-2^. After the PT is overcome, the conductivity starts to increase about 5 orders of the initial value (Figs. 7f, h, j, and l), in accordance with^17^. Similar results can be found in A_2_–A_4_ with a decreasing peak value of TMP, and smaller flat top value of pore density and conductivity, however, TMP of A_5_ exceeds the threshold of 1 V, yet pore density does not overcome the PT and therefore no significant increase in conductivity is observed. Spatial distribution of the TMP, pore density, and conductivity along the arc length of plasma membrane gives similar results, demonstrating that at least 45° near A_1_ of the upper left quarter of the plasma membrane is electroporated, in accordance with^28^. Similar results are also obtained with the application of three other nsPEFs, which are not shown in this paper.

## Discussion and conclusion

This study presents nsPEFs microdosimetric study that includes DP of plasma membrane and nuclear membrane with a second-order Debye model, which has been transformed into the time-domain form with the introduction of polarization vector. Then we obtain the time course of TMP by solving the combination of Laplace equation and time-domain Debye equation. Next, we use the asymptotic version of the Smoluchowski equation to characterize EP and add it to our model to predict the temporal and spatial distribution of TMP and pore density.

During the evaluation of this simulation, we note that it is impossible to find all of the parameters for a single cell. The parameters listed in Table 1, such as cell geometrical size, conductivity, and permittivity of all components, are obtained from external sources, other theoretical models, or experiments. Thus, differences between experimental results and simulation results are predictable. In order to prove the correctness of our simulation, we evaluate the time course of TMP at A_1_ and compared the simulation results with those of the analytical results obtained with the second-order Schwan equation, with the same model parameters listed in Table 1, and our algorithm gives satisfactory accuracy with a maximum difference of about 2%. Induced TMP distribution both in the frequency and time domain is underestimated without considering DP with nsPEFs of frequency spectrum above 10^6^ Hz or pulse duration equals or less than 10 ns, and this trend is in well agreement with previous studies, furthermore, the correctness of the interpretation of Debye model in frequency and the time domain can be proved by spectrum distribution of the relative permittivity and time course of the polarization vector.

One unique aspect of this study is to include both DP and EP in the dielectric double-shelled cell model, to obtain the temporal and spatial distribution of TMP on the plasma membrane without the introduction of complex mathematics. And the algorithm presented in this study can be easily applied to biological cells in irregular shapes, even to real biological cells. Unlike^20^, where EP equation is solved on the surface of plasma membrane and the time-domain Debye equation is solved in the sub-domain of plasma membrane based on the single-shell dielectric cell model, both of them are solved in the sub-domain of the plasma membrane based on the double-shell dielectric cell model, which can be more accurate to quantify TMP and pore density during and after the nsPEFs exposure, as micro-pores are created inside the plasma membrane instead of only the surface. By comparing the simulation results of single-shelled model and double-shelled model, we can intuitively find that the response speed of cell membrane EP under double-shelled model was relatively faster (Fig. 5). Chiapperino et al.^21^ also combined EP and DP in double-shell cells, but they build nuclear membrane in a different way. Two closely contacted thin layers were used to represent the nuclear membrane in their model, while a single material was defined to represent that in our model. The trend of the two results was consistent, but our model was relatively simple.

Another unique aspect of this paper is the comparison of four groups of nsPEF with the equal power density but different polarity and pulse width. In EP mode, TMP of 3 ns and 5 ns bipolar pulses do not reach TMP threshold of 1 V, while TMP of 10 ns and 5 ns unipolar pulses reached 1 V, however, cell is electroporated with only the 5 ns unipolar pulse, as evidenced by the fact that significant increase in pore density and conductivity is observed with only the 5 ns unipolar pulse, which means that only TMP threshold of 1 V is not sufficient to predict the onset of EP of biological cell, time evolution of pore density and (or) conductivity need to be taken into account.

In EP mode, TMP of A_1_–A_7_ follows the cos*θ* law, as evidenced by the peak value of TMP listed in Table. 2, which means that plasma membrane is not electroporated, as previous experimental studies demonstrated that the cos*θ* law is not valid once significant poration occurs, and the results are in accordance with the distribution of pore density and conductivity, where pore density electroporation threshold PT is not overcome and no significant increase in conductivity is observed. In EP + DP mode (Table 2), TMP of different points on plasma membrane does not follow the cos*θ* law. And pore density electroporation threshold PT is overcome in A_1_–A_4_, where significant increase in conductivity is also found, demonstrating that at least 45° of 90° of plasma membrane is electroporated. Krassowska and Filev^28^ found that the boundary of the electroporation and the non-electroporation is 45°, and this value is similar to our simulation results.

In addition, Fig. 7f shows that the location on the membrane closest to the electrodes has the largest pore density, and the pore density decreases from the point to the pole. Pucihar and colleagues^21^ observed that the electrode near the membrane had the maximum fluorescence intensity, which was consistent with our results. Significant increase in conductivity of A_1_–A_4_ of about 5 orders is observed in Fig. 7j, in agreement with^29^, in which conductivity of an oxidized cholesterol membrane with the application of 20 μs pulse was measured, and significant increase of 4 to 5 orders in conductivity was found. Although previous studies showed that nsPEFs induced more pronounced increase in conductivity through EP than that of μsPEFs^13^, our simulation can give comparable results.

In this study, only dielectric relaxation of plasma membrane and nuclear membrane are included, however, dielectric relaxation of the extracellular medium and cytoplasm has to be included when spectrum of PEF exceeds 20 GHz^16^. The pore radius which is considered constant in this paper actually varies with time and space and needs to be considered in a more detailed model which introduces the pore radius variation equation^28^. Furthermore, biological cells with irregular shape or real cells should be modeled instead of a spherical cell. These factors will be considered in our future studies.
